# Identification of Aloperine as an anti-apoptotic Bcl2 protein inhibitor in glioma cells

**DOI:** 10.7717/peerj.7652

**Published:** 2019-09-03

**Authors:** Zhijie Xu, Xiang Wang, Xi Chen, Shuangshuang Zeng, Long Qian, Jie Wei, Zhicheng Gong, Yuanliang Yan

**Affiliations:** 1 Department of Pathology, Xiangya Hospital, Central South University, Changsha, China; 2 Department of Pharmacy, Xiangya Hospital, Central South University, Changsha, China; 3 National Clinical Research Center for Geriatric Disorders, Xiangya Hospital, Central South University, Changsha, China

**Keywords:** Bcl2 inhibitor, Apoptosis, Aloperine, Glioma

## Abstract

**Objective:**

Aloperine (ALO), an alkaloid isolated from the leaves of *Sophora alopecuroides*, has been suggested to exhibit anti-inflammatory and anti-tumor properties and is traditionally used to treat various human diseases, including cancer. However, limited information is available about the mechanisms that determine the anti-tumor activities of ALO.

**Methods:**

Herein, through comprehensive bioinformatics methods and in vitro functional analyses, we evaluated the detailed anti-tumor mechanisms of ALO.

**Results:**

Using the databases Bioinformatics analysis tool for molecular mechanism of traditional Chinese medicine and PubChem Project, we identified the potential targets of ALO. A protein–protein interaction network was constructed to determine the relationship among these probable targets. Functional enrichment analysis revealed that ALO is potentially involved in the induction of apoptosis. In addition, molecular docking demonstrated that ALO expectedly docks into the active pocket of the Bcl2 protein, suggesting Bcl2 as a direct target of ALO. Moreover, western blot and qPCR analysis showed that ALO downregulated Bcl2 expression in human glioma cell lines, SK-N-AS and U118. Using flow cytometry methods, we further confirmed that ALO significantly promotes apoptosis in SK-N-AS and U118 cell lines, similar to the effect induced by ABT-737, a well-known Bcl2 inhibitor. In addition, Bcl-2 overexpression could rescue ALO-induced Bcl-2 inhibition and suppress pro-apoptotic effects in glioma cells.

**Conclusion:**

Taken together, these findings suggest that the natural agent ALO effectively enhances apoptosis by acting as a potential Bcl2 inhibitor in human glioma cells.

## Introduction

Current cancer treatment paradigms, combining radiotherapy and chemotherapy with cytotoxic drugs, are frequently restricted by dose-limiting toxicity and severe adverse effects. Therefore, identifying novel, safe chemo-adjuvants from herbal medicine that selectively and effectively enhance the effects of chemotherapeutic agents may potentially convey improved treatment for cancer (Jin et al., 2012). Given the ample chemical diversity and plethora of molecular targets, several natural products, such as triptolide, diosmetin, tanshinone and others, are poised to become essential sources for new anti-tumor agents (Wei et al., 2019; Xu et al., 2017; Yan et al., 2018). Aloperine (ALO) is a kind of alkaloid with a unique endocyclic scaffold extracted from the Chinese natural product *Sophora alopecuroides*. Heretofore, ALO has been reported to exert therapeutic effects against pulmonary hypertension, renal injury and colon cancer (Zimecki, 1987). ALO derivatives with a unique endocyclic scaffold have also been designed, synthesized and evaluated as HCV, PD-L1 and HIV-1 entry inhibitors (Dang et al., 2016; Zhang et al., 2019). Moreover, analysis demonstrated that ALO suppresses tumorigenesis, suggesting its potential therapeutic use in human cancers and multidrug-resistant cancers.

The anti-tumor effects of ALO have been demonstrated in several human cancer cells, including colon cancer, lung cancer and multiple myeloma. One study showed that ALO induces G2/M phase cell cycle arrest with concomitant alterations in p21, p53, cyclin D1 and cyclin B1. In addition, ALO inhibits phosphatidylinositol 3-kinase/Akt and JAK/Stat3 pathways, inducing apoptosis in colon cancer cells (Zhang et al., 2014). ALO exerts its anti-tumor effects by attenuating oxidative stress and promoting dual apoptotic mechanisms. Targeting cFLIP and phosphorylated-PTEN, ALO induces apoptosis in multiple myeloma cells through both the intrinsic and extrinsic apoptotic pathways (Wang et al., 2015). ALO also inhibits cell growth through the induction of caspase-dependent apoptosis in human anaplastic thyroid cancers and multidrug-resistant papillary thyroid cancers (Lee et al., 2018). Through suppression of the PI3K/AKT signaling pathway, ALO induces apoptosis and inhibits invasion in human osteosarcoma cells (Chen et al., 2018). By blocking Ras signaling, ALO inhibits proliferation, migration and invasion, inducing apoptosis in human breast cancer cells (Tian et al., 2018). As an anti-inflammatory agent, studies have shown that ALO suppresses allergic airway inflammation through NF-κB, MAPK and Nrf2/HO-1 signaling pathways in asthmatic mice (Wang et al., 2018). However, Ren et al. found that ALO treatment significantly reduces H_2_O_2_-induced caspase-9 activity in nucleus pulposus cells. The mechanism of ALO’s protective effects occurs via anti-apoptotic activity and suppression of the NF-κB signaling pathway (Ren et al., 2017). These results suggest that ALO function is variable in distinct disease models and is worthy of further investigation as an agent with chemotherapeutic activity in cancer research.

Here, we performed bioinformatics screening to identify novel ALO targets, subsequently identifying Fas, caspase 3 (Casp3), Bcl2, C-X-C motif chemokine receptor 4 (CXCR4) and solute carrier family 6 member 2 (SLC6A2) as potential targets for ALO. Using gene ontology (GO) and Kyoto Encyclopedia of Genes and Genomes (KEGG) analysis, as well as protein–protein interaction (PPI) software, we also studied the role and pathways affected by ALO. Using the Systemsdock platform for molecular docking analysis, we observed that ALO expectedly docked into the active pocket of the Bcl2 structure, suggesting that Bcl2 is a direct target through which ALO exerts its anti-tumor activities. Several studies have demonstrated that ALO causes cell death and apoptosis in solid tumors. In this study, we suggest that Bcl2 inhibition by ALO induces apoptosis in glioma cells. In addition, ALO’s inhibitory effect was compared to the Bcl2 inhibitor, ABT199.

## Materials and Methods

### Data acquisition and analysis using different bioinformatics methods

Bioinformatics analysis for drug target discovery was performed using several public web-based tools, which are summarized in Table S1.

The bioinformatics analysis tool for molecular mechanism of traditional Chinese medicine (BATMAN-TCM) is a user-friendly online tool to evaluate the functions of TCM in human pathophysiological processes (Liu et al., 2016). PubChem is a chemical information resource for biomedical research (Kim et al., 2019). Using these two algorithms, we screened possible molecular targets of the natural compound ALO. After PPI network of these targets was constructed by STRING v11 (Szklarczyk et al., 2019), we performed GO and KEGG pathway enrichment analysis by WEB-based Gene Set Analysis Toolkit (WebGestalt) (Wang et al., 2017) and FunRich (Pathan et al., 2015), respectively. Next, molecular docking was conducted by systemsDock (Hsin et al., 2016) to evaluate interactions between ALO and its predicted targets. Protein sequence alignment of Bcl2 Arg63–Ala108 was conducted in Clustal Omega to identify sequence divergence.

### Cell culture

Glioma cell lines, including T98G, U87, U118, SK-N-AS, U251 and Hs683, were obtained from the Cancer Research Institute, Central South University, China. Glioma cells were maintained in Dulbecco’s modified Eagle’s medium (DMEM; Gibco, Invitrogen, Carlsbad, CA, USA) with 10% fetal bovine serum (Gibco, Invitrogen, Carlsbad, CA, USA) and 100 U/ml penicillin-streptomycin (Gibco, Invitrogen, Carlsbad, CA, USA) at 37 °C and 5% CO_2_.

### Reagents and plasmids

Aloperine was purchased from Selleck Chemicals (S2420). The Bcl2 inhibitor ABT199 was purchased from MedChemExpress (HY-15531). All reagents were dissolved in dimethylsulfoxide (Amresco, Solon, OH, USA). Bcl2 over-expression plasmid was purchased from MiaoLingBio (P8816).

### RNA extraction, cDNA synthesis and real-time PCR

Total RNA from renal tissues was isolated using Trizol reagent (Invitrogen, San Diego, CA, USA). Total RNA was then reverse-transcribed to cDNA using the PrimeScript™ strand cDNA synthesis kit (6210; Takara, Dalian, China). Densitometry was analyzed using 200TM-Image software (Bio-Rad, Hercules, CA, USA), and actin served as an internal reference gene. Forward and reverse primer sequences were as follows: Actin: 5′-CATGTACGTTGCTATCCAGGC-3′ and 5′-CTCCTTAATGTCACGCACGAT-3′; Bcl2: 5′-GGTGGGGTCATGTGTGTGG-3′ and 5′-CGGTTCAGGTACTCAGTCATCC-3′. Expression levels were evaluated using iTaqTM Universal SYBR green Supermix (Bio-Rad, Hercules, CA, USA) under the following conditions: 95 °C for 5 min, 40 cycles of 95 °C for 15 s, 56 °C for 30 s. Data analysis was conducted using the 2^−ΔΔCt^ method.

### Western blot

Briefly, approximately 5 × 10^6^ glioma cells were washed in PBS and lysed in lysis buffer (Thermo Scientific, Waltham, MA, USA). Then, lysates were incubated on ice for 30 min and centrifuged at 10,000×*g* for 30 min at 4 °C. Protein concentrations were calculated by a BCA kit (Thermo Scientific, Waltham, MA, USA). After sample separation by 15% SDS-PAGE, proteins were transferred onto NC membranes (HATF00010; Burlington, MA, Millipore). Membranes were incubated overnight at 4 °C for 1 h after blocking with primary antibodies as follows: Bcl2 (1:1,000, #2872; Cell Signaling Technology, Danvers, MA, USA) and tubulin (1:2,000; Proteintech, Chicago, IL, USA). Then, membranes were probed with secondary antibodies (1:10,000; Proteintech, Chicago, IL,USA). Bands were detected using Immobilon western chemiluminescent reagents (WBKLS0500; Millipore, Burlington, MA, USA).

### Apoptosis analysis

Apoptosis assays were conducted using the Annexin V-fluorescein isothiocyanate apoptosis kit (BD Biosciences, San Jose, CA, USA) according to the manufacturer’s protocols. The results were analyzed with a Dxp Athena™ flow cytometer (Cytek, Fremont, CA, USA).

### Statistical analysis

All data in this study were presented as means ± standard deviations and experiments mentioned above were repeated at least three times. The significance of difference among the means was calculated using *t*-tests for two groups using SPSS 19.0 software. Statistical significance was defined as *p* < 0.05 in all tests.

## Results

### Prediction of potential ALO targets using online bioinformatics analysis tools

**Figure 1:**
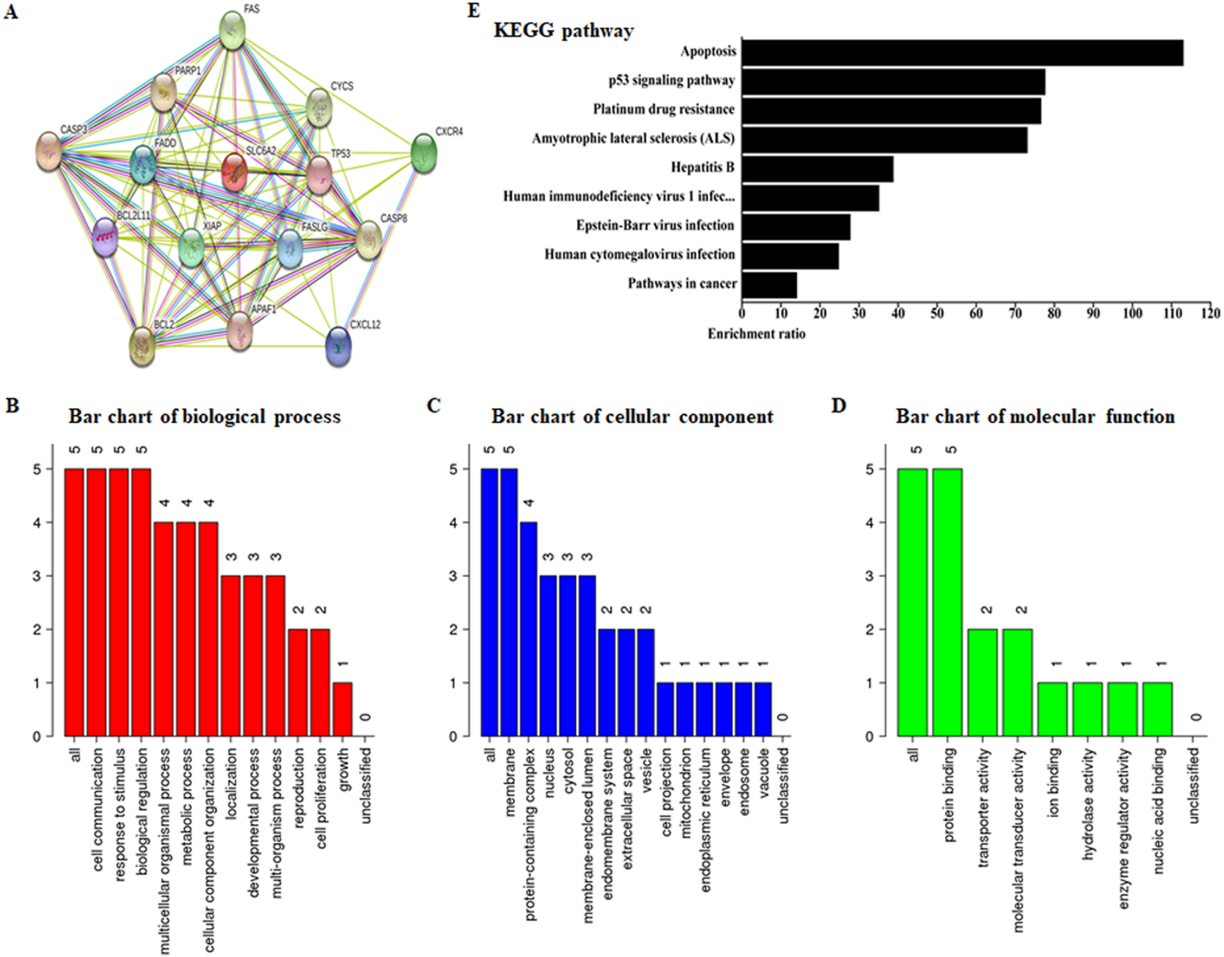
Functional enrichment and interaction network analysis of potential molecular targets of ALO. (A) Protein interaction network was constructed using STRING v11. (B–D) GO and (E) KEGG pathway enrichment analysis by WebGestalt and FunRich, respectively.

**Figure 2:**
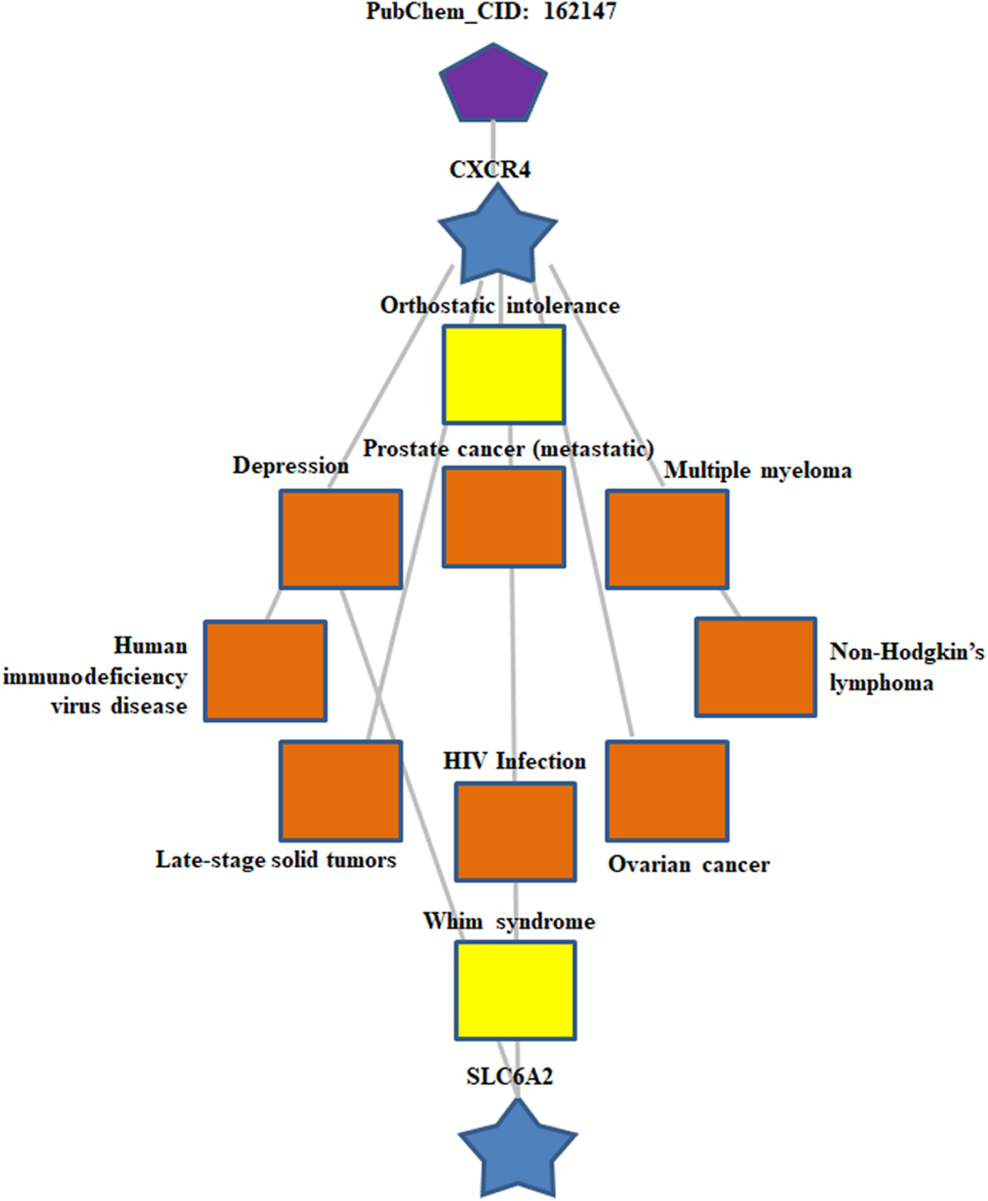
The functional roles of ALO in human diseases. Data from BATMAN-TCM revealed that ALO shows significant therapeutic effects on several human pathological processes, especially human cancers.

Two public bioinformatics databases, BATMAN-TCM and PubChem Project, were used to identify potential targets of the natural compound ALO. As shown in Table 1, we observed that Fas, Casp3, anti-apoptosis protein Bcl2, CXCR4 and SLC6A2 were potential molecular targets of ALO. To assess the involvement of these targets in the biological behaviors of human biology, PPI for these five genes was conducted by a frequently used algorithm, STRING v11 (Fig. 1A). Furthermore, the WebGestalt database was used for analyzing GO enrichment of these five genes. Interestingly, several well-known processes involved in human pathology and physiology were identified in our top-ranked GO processes, including cell communication and response to stimulus (ontology: biological process), membrane and protein-containing complex (ontology: cellular component), protein binding (ontology: molecular function) (Figs. 1B–1D). Significant pathways of these targeted genes were compared using KEGG analysis to further illuminate their function. KEGG pathway analyses by FunRich proteomics software revealed that apoptosis was obviously enriched among identified biological signaling pathways (*p*-value < 0.01 was recommended as the cut-off) (Fig. 1E). Next, according to data from BATMAN-TCM, we evaluated the roles of ALO in human disease. Expectedly, ALO showed significant therapeutic effects on several human cancer and other pathological processes, such as late-stage solid tumor (Fig. 2; Table 2). Taken together, these findings suggest that agent ALO might exert anti-tumor effects by inducing cell apoptosis.

**Table 1:** Predicted targets of Aloperine by two tools, BATMAN-TCM and PubChem.

Targets	Description	Location	Aliases
Fas	Fas cell surface death receptor	Chromosome 1	Tnfrsf6
Casp3	caspase 3	Chromosome 16	CPP32-beta, Lice, Yama
Bcl2	BCL2, apoptosis regulator	Chromosome 13	Bcl2
CXCR4	C-X-C motif chemokine receptor 4	Chromosome 2	CD184, D2S201E, FB22, HM89
SLC6A2	solute carrier family 6 member 2	Chromosome 16	NAT1, NET, NET1, SLC6A5

**Table 2:** The human diseases associated with ALO treatment.

Diseases	*p*-value
Late-stage solid tumors	1.34E-02
HIV Infection	1.34E-02
Prostate cancer (metastatic)	1.49E-02
Non-Hodgkin’s lymphoma	1.56E-02
Multiple myeloma	1.60E-02
HIV disease	2.79E-02
Depression	2.79E-02
Ovarian cancer	3.10E-02
Breast cancer	6.35E-02

### Molecular docking identifies Bcl2 as a target of ALO

**Figure 3:**
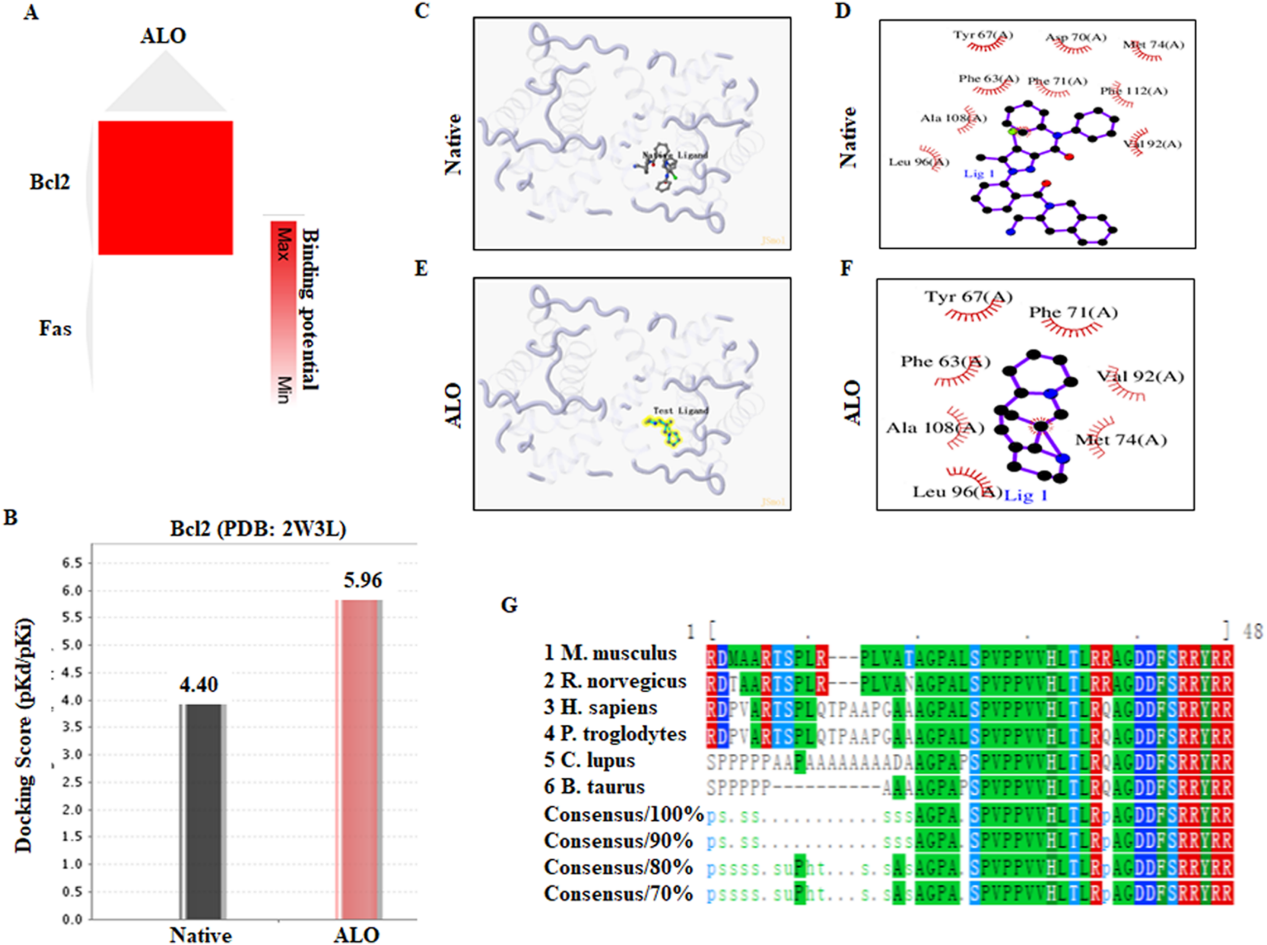
Docking positions and an activity pocket for ALO in the crystal structure of Bcl2. (A) A heatmap indicating the binding potential between ALO and its targets in homo species. (B) The docked energy value between ALO and the Bcl2 protein structure. (C–F) Important residues are labeled and shown as sticks to facilitate localization of native ligand or ALO on the active site region in Bcl2. (G) The phylogenetic analysis using the Clustal Omega tool for Arg63–Ala108 in Bcl2 sequence between homo sapiens and other species.

By analyzing the macromolecular structure archive from the RCSB Protein Data Bank (PDB) (Burley et al., 2017), we confirmed preferred 3D protein structures of ALO target molecules (Fas: 3HHD, Casp3: 2DKO and Bcl2: 2W3L) in homo species. However, no 3D shape of CXCR4 and SLC6A2 proteins could be reliably generated in the PDB database. Then, using the molecular docking platform systemsDock, we detected protein-ligand binding profiles between ALO and these potential targets. As shown in Figs. 3A and 3B, we found that ALO could be well docked into the 3D protein structure of Bcl2, with the highest docking score values of 5.960 pKd/pKi. Next, we evaluated the possible activity pocket for ALO within the Bcl2 protein. The ligand-receptor binding pattern revealed that the native ligand could be docked into the Phe63–Val192 domain of Bcl2 in homo species, indicating the Phe63–Val192 domain is the active binding pocket of Bcl2 protein. Moreover, we found that ALO was also docked into the consistent pocket as expected, which includes amino acids Phe63, Tyr67, Phe71, Met74, Val92, Leu96 and Ala108 (Figs. 3C–3F). In addition, the phylogenetic analysis using the Clustal Omega tool (Madeira et al., 2019) revealed that the Bcl2 is highly conserved from homo sapiens to other species, with several conservative amino acid sites, particularly Val92–Ala108 in homo sapiens, suggesting its important function in biological processes (Fig. 3G). Thus, these data indicate good affinity of ALO for the Bcl2 protein that occurs by forming intermolecular forces between their polar moieties and conservative amino acid residues.

### ALO induces apoptosis via Bcl2 inhibition in glioma cells

**Figure 4:**
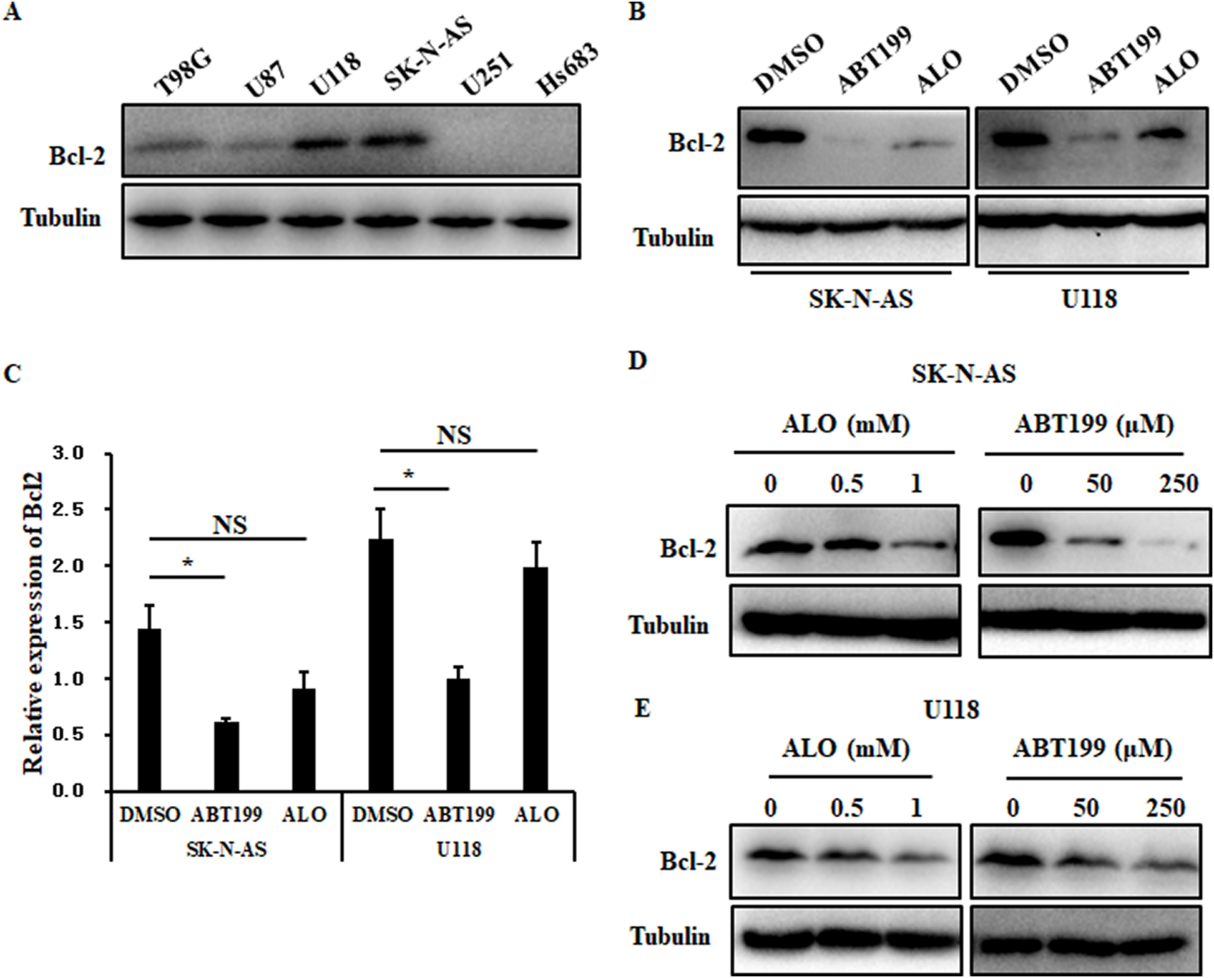
ALO reduces Bcl2 protein expression in glioma cells. (A) Expression of Bcl2 was analyzed by Western blot in glioma cell lines, including T98G, U87, U118, SK-N-AS, U251 and Hs683. Western blot (B) and real-time PCR (C) were used to analyze Bcl2 expression in response to one mM ALO and 250 μM ABT199 in SK-N-AS and U118 cells. (D) and (E) Western blot analysis of Bcl2 expression after gradient concentrations of ALO (0, 0.5, 1 mM) and ABT199 (0, 50, 250 μM) treatment. The experiments were repeated for three independent times. The quantitative results shown are means ± SD. An asterisk (*) indicates the significant difference. NS indicates no significant difference.

**Figure 5:**
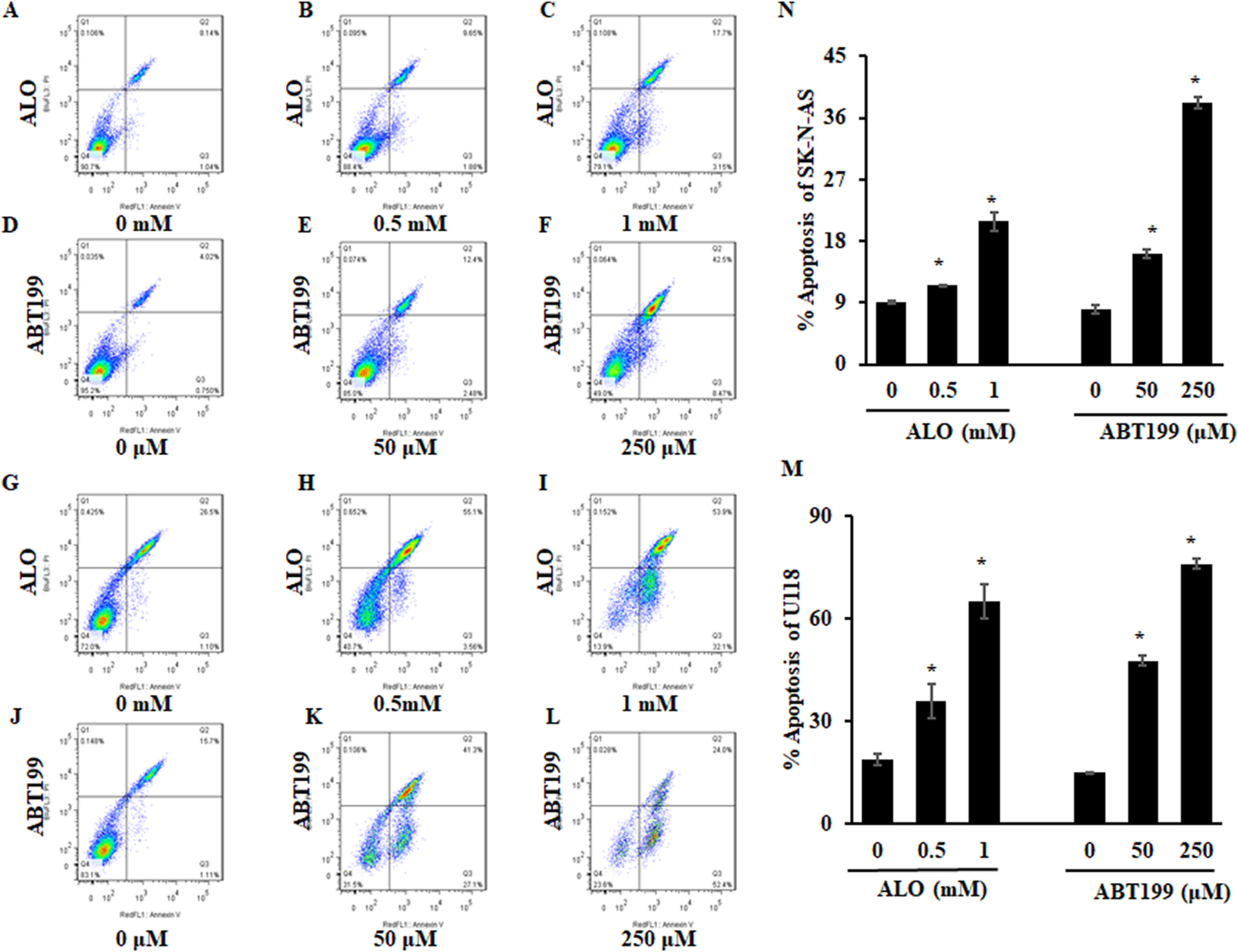
ALO induces apoptosis in glioma cells. Flow cytometry results (A–L) and apoptosis rate (N) and (M) after gradient concentration of ALO (0, 0.5, 1 mM) and ABT199 (0, 50, 250 μM) treatment in SK-N-AS and U118 cells. The experiments were repeated for three independent times. The quantitative results shown are means ± SD. An asterisk (*) indicates the significant difference. NS indicates no significant difference.

**Figure 6:**
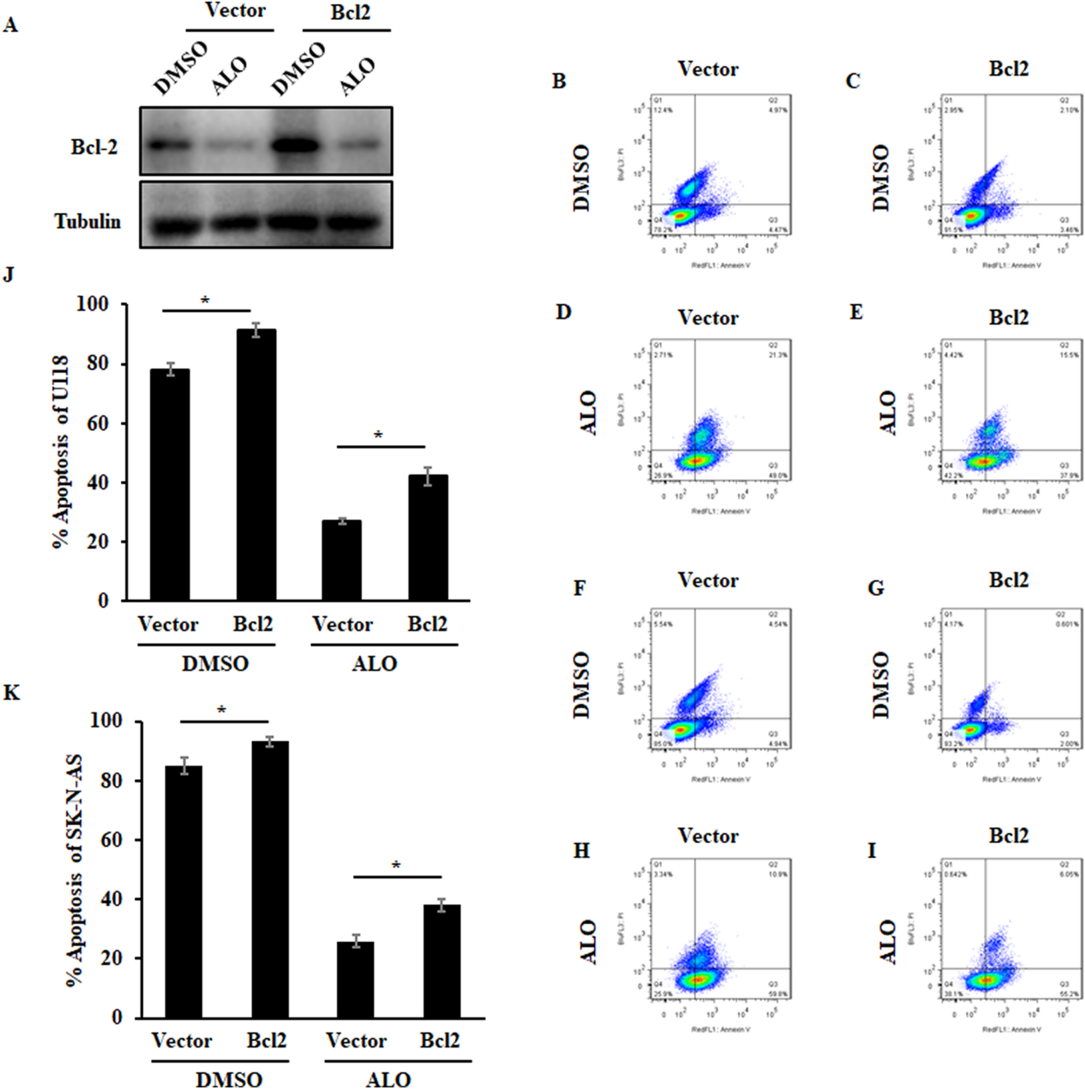
Over-expressed Bcl2 suppresses the pro-apoptosis effect of ALO in glioma. (A) Glioma cells were treated with one mM ALO and Bcl2 over-expression plasmid single or combined, respectively. Then the Expression of Bcl2 was analyzed by Western blot. Flow cytometry results (B–I) and apoptosis rate (J) and (K) were analyzed after indicated treatment in SK-N-AS and U118 cells. The experiments were repeated for three independent times. The quantitative results shown are means ± SD. An asterisk (*) indicates the significant difference. NS indicates no significant difference.

Glioma, a highly invasive and fatal cancer, is one of the most highly malignant human solid tumors, accounting for approximately 80% of all primary brain tumors (Chen et al., 2019). Studies have shown that natural compounds provide a large source of bioactive compounds with excellent therapeutic efficacy and have been investigated for their anticancer properties (Xu et al., 2018). After determining the relationship between ALO and solid tumors, we further investigated the effect of ALO on the inhibition of Bcl2 in glioma cells. Protein levels of Bcl2 were obviously higher in U118 and SK-N-AS compared to T98G, U87, U251 and Hs683 glioma cells (Fig. 4A). We then assessed the effect of ALO on Bcl2 compared to the Bcl2 inhibitor ABT199. After treatment with ALO and ABT199, we observed that protein levels of Bcl2 were obviously reduced in both groups, while mRNA expression in the ALO group was not significantly reduced in U118 and SK-N-AS cells (Figs. 4B and 4C). Using a concentration gradient, expression of Bcl2 consistently declined in response to treatment with 0.5 and one mM ALO or 50 and 250 μM ABT199 in glioma cells (Figs. 4D and 4E). Pathways regulated by Bcl2 play key roles in apoptosis, including controlling mitochondrial membrane permeability, forming feedback loop systems with caspases and attenuating inflammation (Kontos, Christodoulou & Scorilas, 2014). To investigate whether Bcl2 inhibition caused by ALO is associated with cell apoptosis in glioma cells, we examined the apoptosis rate in response to ALO and ABT199 treatment. Flow analysis indicated consistently induced apoptosis in U118 and SK-N-AS cells, further supporting the phenotype of Bcl2 inhibition (Fig. 5). To further confirm the targeting relationship between ALO and Bcl2, the effects of ALO and Bcl2 over-expression plasmid single or combined treatment were analyzed in glioma cells. Western blot experiments showed ALO-induced Bcl2 inhibition was significantly decreased in combination treatment (Fig. 6A). Additionally, over-expressed Bcl2 suppressed the pro-apoptosis effect of ALO in glioma (Figs. 6B–6K).

## Discussion

In this study, we investigated possible molecular targets of ALO, demonstrating that the natural compound ALO exerts obvious anti-tumor activity by inducing apoptosis in human glioma cells. In our mechanism analysis, ALO seems to directly dock into the active pocket of the Bcl2 protein, weakening Bcl2-mediated anti-apoptotic effects.

Over the past decade, several reports have demonstrated that ALO is a promising therapeutic drug for the treatment of human diseases, including cancers. A better understanding of the mechanism of ALO, particularly in human cancer patients, represents a matter of great interest for probable clinical practice in the future. The results from Dang’s group showed that ALO administration inhibits immunodeficiency virus-1 (HIV-1) infection by impeding viral entry (Dang et al., 2016). By optimizing the structure of ALO, Dang et al. (2017) identified an ALO derivative with an approximately 15-fold increase in anti-HIV-1 activity. As for its anti-cancer roles, ALO induces anti-tumor effects against several human cancers, including multiple myeloma, prostate cancer, breast cancer, thyroid cancer, osteosarcoma and hepatocellular carcinoma (Chen et al., 2018; Lee et al., 2018; Ling et al., 2018; Liu et al., 2019; Tian et al., 2018; Wang et al., 2015). However, no specific data shows that ALO impacts apoptosis related genes in glioma. In addition, ALO suppresses cell proliferation and invasion in human osteosarcoma cell lines, MG-63 and U2OS, by down-regulating the protein kinase B (Akt) signaling pathway (Chen et al., 2018). ALO induces cell-cycle arrest and apoptosis in HCT116 human colon cancer cells via cosuppression of Akt and signal transducer and activator of transcription 3 (STAT3) signaling pathways (Zhang et al., 2014). Based on these findings, as shown in Fig. 2 and Table 2, we found that ALO has significant effects on several human pathological behaviors, such as HIV infection and cancers. Then, using two glioma cell lines, SK-N-AS and U118, we further confirmed that ALO significantly promotes apoptosis by reducing Bcl2 mRNA and protein expression levels.

Since the elucidation of apoptosis in programed cell death, anti-apoptotic proteins have been a model for generating small-molecule inhibitors. With innovative medicinal chemistry and structure-based drug design, Bcl2-selective inhibitors, such as venetoclax, have been discovered in recent decades (Leverson et al., 2017). A preclinical study demonstrated that venetoclax, a potent and selective Bcl2 inhibitor recently approved by the FDA and the European Medicines Agency, combined with endocrine therapy, exerted a tolerable safety profile and elicited notable activity in estrogen receptor and Bcl2-positive metastatic breast cancer (Lok et al., 2019). Further studies suggest that a Bcl2 inhibitor, ABT-737, reverses drug resistance and increases sensitivity to prednisolone in the treatment of early T-cell precursor-acute lymphoblastic leukemia (Kawashima-Goto et al., 2015). In Phase 1 studies in patients with relapsed or refractory CD20+ lymphoid malignancies, the Bcl2 inhibitor navitoclax combined with rituximab displayed essential clinically activity by safety, pharmacokinetics, and anti-tumor activity analyses (Roberts et al., 2015). Moreover, novel small molecular compounds acting as Bcl2 inhibitors have been discovered to induce apoptosis and inhibit cell viability. Vartak et al. (2017) found that Disarib predominantly binds to the BH1 domain and specifically disrupts the Bcl2-BAK interaction in in vitro biochemical and biophysical analyses (Iyer et al., 2016). Using mouse allograft and xenograft models, the same teams showed that Disarib administration causes tumor regression and exhibits no significant side effects (Vartak et al., 2016).

Naturally occurring compounds are an infinite treasure of bioactive chemicals and an inexhaustible resource for discovery. Until now, few bioactive chemicals have been reported that have anti-apoptotic Bcl-2-inhibiting activity against solid or other human tumors. Astragalin, a naturally occurring flavonoid, has been discovered in plants such as *Cuscuta chinensis* Lam. Riaz et al. (2018) showed that astragalin functions include the regulation and modulation of various molecular targets, such as transcription factors, enzymes, kinases, cell adhesion proteins, inflammatory cytokines and apoptosis proteins (such as anti-apoptotic Bcl-2). Using molecular docking, several bioactive compounds from *Annona muricata* Linn were shown to be potential inhibitors of anti-apoptotic proteins, such as Bcl-2, Bcl-w and Mcl-1 (Mohamad Rosdi et al., 2018). In a structure-based virtual ligand screening, 8-chrysoeriol, a bioactive flavonoid, was identified to bind directly to Bcl-2, similar to BH3 mimetics, and to trigger apoptosis in pancreatic cancer cells (Zhang et al., 2018). Our results verified that ALO could target the anti-apoptotic protein Bcl-2 in glioma cells but not in other cancer cells. The results of this study suggest that the anti-tumor activities of ALO need to be further investigated in other solid human tumors.

## Conclusions

We successfully identified and characterized the potential anticancer compound, ALO, which enhances apoptosis in human glioma cells. The identification of natural compounds that serve as Bcl2 inhibitors, such as ALO, might prove to be promising for the eradication of glioma cells.

## Supplemental Information

10.7717/peerj.7652/supp-1 Supplemental Information 1 The main bioinformatics databases used to analyze the roles of ALO in the cancer biology.

10.7717/peerj.7652/supp-2 Supplemental Information 2 Raw data.

## Additional Information and Declarations

### Data Availability

The following information was supplied regarding data availability: The raw data are available in the Supplemental Files.
