# Supplementary material for: Identification of Aloperine as an anti-apoptotic Bcl2 protein inhibitor in glioma cells
Source: PeerJ. 2019 Sep 3;7:e7652. doi: 10.7717/peerj.7652 (PMC6730530; doi:10.7717/peerj.7652)
Supplement: Supplemental Information 1 [file peerj-07-7652-s001.docx]

Table S1. The main bioinformatics databases used to analyze the roles of ALO in the cancer biology.

| Databases | URL | Refs |
| --- | --- | --- |
| BATMAN-TCM | http://bionet.ncpsb.org/batman-tcm | (Liu et al., 2016) |
| PubChem | https://pubchem.ncbi.nlm.nih.gov | (Kim et al., 2019) |
| STRING | https://string-db.org/ | (Szklarczyk et al., 2019) |
| WebGestalt | http://www.webgestalt.org/option.php | (Wang et al., 2017) |
| FunRich | http://www.funrich.org | (Pathan et al., 2015) |
| Systemsdock | http://systemsdock.unit.oist.jp/ | (Hsin et al., 2016) |
| PDB | https://www.rcsb.org/ | (Burley et al., 2017) |
